# IMPACT OF MULTIPLE CHRONIC CONDITIONS ON CHANGE IN PHYSICAL FUNCTION FROM MID- TO EARLY LATE LIFE

**DOI:** 10.1093/geroni/igz038.1985

**Published:** 2019-11-08

**Authors:** Brittney S Lange Maia, Kelly Karavolos, Elsa Strotmeyer, Carrie Karvonen-Gutierrez, Elizabeth Avery, Imke Janssen, Sheila Dugan, Howard Kravitz

**Affiliations:** 1 Rush University Medical Center, Chicago, Illinois, United States; 2 University of Pittsburgh, Pittsburgh, Pennsylvania, United States; 3 University of Michigan, Ann Arbor, Michigan, United States

## Abstract

Chronic conditions emerging in midlife may be modifiable to prevent progression ultimately preserve physical function (PF) in late life. We quantified change in perceived PF in relation to several common chronic conditions known to impact PF in late life (osteoarthritis, diabetes, stroke, hypertension, heart disease, cancer, osteoporosis, and depression). Physical function (PF) was assessed using the Physical Functioning Scale of the SF-36 among 2,283 women in SWAN from an average age of 50.0±2.7 to 64.0±3.7 years. In covariate-adjusted Poisson models, each additional condition was associated with 3% worse PF (p<0.001), and an additional 0.4% annual worsening (p<0.001). Thus, holding demographic, lifestyle, socioeconomic, and other health factors constant, a woman a decade later entering old age with no chronic conditions would have 8.1%, 15.5%, and 17.0% better PF vs. having one, two, or three conditions, respectively. Preventing or delaying chronic disease progression in midlife may improve PF trajectories into late life.

